# Dr. John Edward Meredith

**Published:** 1912-09-14

**Authors:** 


### Obituary.

Dr. John Edward Meredith, of Maidstone, has died after a long illness at St. Leonards. He was a very familiar figure in the Kentish county town, being public vaccinator and district medical officer. Dr. Meredith served for many years as honorary physician to the West Kent General Hospital, Maidstone, and at the time of his death he was consulting physician to this institution. He received his medical education at Trinity College, Dublin, where he qualified in 1872, later taking the degrees of M.D. and M.Ch.

